# Evaluation of light-emitting diode colors and intensities on slaughter performance, meat quality and serum antioxidant capacity in caged broilers

**DOI:** 10.5713/ab.22.0193

**Published:** 2022-11-13

**Authors:** Zichao Tan, Chuanfeng Zhou, Xueping Shi, Lihua Wang, Shubai Wang

**Affiliations:** 1 College of Animal Science and Technology, Qingdao Agricultural University, Qingdao 266109, China; 2 College of Life Science, Qingdao Agricultural University, Qingdao 266109, China

**Keywords:** Color, Intensity, Light-emitting Diode, Meat Quality, Serum Antioxidant Capacity, Slaughter Performance

## Abstract

**Objective:**

This study was to evaluate the interaction of three different light-emitting diode (LED) light colors (white, green, and blue) and three intensities (5, 10, and 15 lx) on slaughter performance, meat quality and serum antioxidant capacity of broilers raised in three-layer cages.

**Methods:**

A total of 648 (8-days-old) male broiler chicks (Cobb-500) were randomly assigned in 3×3 factorially arranged treatments: three light colors (specifically, white, blue, and green) and three light intensities (namely, 5, 10, and 15 lx) for 35 days. Each treatment consisted of 6 replicates of 12 chicks. The test lasted for 35 days.

**Results:**

The semi-eviscerated weight percentage (SEWP) in 5 lx white was higher than that in 15 lx (p<0.01). The eviscerated weight percentage (EWP) (p<0.05) and water-loss percentage (WLP) (p<0.01) decreased in 10 lx white light than those in green light. Under blue light, the content of hypoxanthine (Hx) in muscle was lower than that under white and green light (p<0.01). The content of malondialdehyde (MDA) in 15 lx blue light was higher than that in 10 lx green light (p<0.05). Light color had an extremely significant effect on thigh muscle percentage, WLP, Hx, and crude protein content (p<0.01). Light intensity had a significant effect on SEWP (p<0.05), EWP (p<0.05), lightness (L*) value (p<0.05), WLP (p<0.01), and the contents of superoxide dismutase (p<0.05), MDA (p< 0.01), glutathione peroxidase (p<0.01).

**Conclusion:**

Using white LED light with 10 lx light intensity can significantly improve the chicken quality of caged Cobb broilers, improve the content of inosine acid in chicken breast and enhance the antioxidant capacity of the body. We suggest that the broiler farm can use 10 lx white LED light source for lighting in 8 to 42 days.

## INTRODUCTION

For decades, poultry industrialization has been developing rapidly. Broiler meat has become the most consumed meat in the world [1]. Poultry industrialization has promoted the productivity of many countries and produced significant economic value [2]. The intensive production system of broiler is widely used in the world due to its strong practicability, high utilization rate and high return on investment.

The importance of lighting regimen is increasing with the industrialization of poultry production [3]. In an environmentally controlled chicken house, the only light source for chicks is artificial supplementary light [4]. The visual response of broilers is sensitive, light hits broiler skull at the retinal receptors and traveling through neurons to the pineal gland, stimulating pineal gland, and hypothalamus regulating functions including metabolism and reproduction [5]. Light regulates the body’s biologic systems in many ways. Its effect on the systemic immune response suggests that it is important in maintaining health, as well as in the induction of disease [6].

Presently, the light source used in broiler houses is mainly fluorescent lighting. With the continuous rise of energy prices and the desire for environmental protection, people are more and more interested in using less energy consumption and more environmentally friendly light sources [7]. Compared with 8,000 hours of fluorescent lighting, the lighting time of light-emitting diode (LED) can reach 100,000 hours. Besides, with the development of LED lighting technology, which has the advantages of high luminous efficiency, low energy consumption, and can be customized according to the characteristics of light consumption, it has received more and more attention in modern broiler farms [4].

Light color, intensity and source are important components of the light environment that affects broiler growth, activity, and welfare [8]. Long wavelengths are known for higher penetration power compared to short wavelengths [9]. In the past few years, many scholars have studied the effects of light on performance, meat quality and welfare of broilers. The present studies have indicated that appropriate light for broilers can help to increase the growth performance, immunity functions, metabolism, behavior, and welfare, ameliorate the systematic immune response, metabolism, and welfare. Olanrewaju et al [10] studied the effects of four light sources (incandescent lamp, compact fluorescent lamp, neutral led, special filter led for cold poultry) and two light intensities (5 lx, 20 lx) on broilers. The results showed that the light source and light intensity had no effect on breast meat weight and yield and would not damage the welfare of heavy broilers. Compared to red light, both the mechanical barrier and immunological barrier were improved in the small intestine of broilers by green light in the early growth stage and by blue light in the later growth stage [11]. The growth and production performance of broilers increased under green light in the early stage (0 to 26 days old) or blue light in the late stage (27 to 49 days old). Blue and green monochromatic light can more effectively stimulate testosterone secretion, thus promoting the growth of muscle fibers [12]. Broilers reared under blue or green light were significantly heavier than those reared under red or white light and green light stimulated growth of chicks at early age [13]. Mohamed et al [14] showed that monochromatic blue light could improve broilers performance and welfare than white light, blue light could increase the final body weight (BW), reduced respiratory rate, heterophils to lymphocytes ratio and interlukien-1β.

Therefore, this study aimed to investigate the influence of lighting color as white, green, and blue LED and intensities (5, 10, and 15 lx) on the slaughter performance, meat quality and serum antioxidant capacity of multi-layer caged broiler chicks.

## MATERIALS AND METHODS

The experiment was performed in a layer unit. Housing, management, and care of birds conformed to standard feeding guidelines of Animal Research Committee of the Qingdao Agricultural University, Qingdao, China (No. 027/2020).

### Husbandry, diets, and experimental design

A total of 648 (8-days-old) Cobb broiler chicks (obtained from Shandong Yisheng Livestock & Poultry Breeding Co., Ltd., Shandong, China) with a similar initial weight (226.40 ±8.67 g) were used in a completely randomized design and were randomly allocated to one of the following 9 groups. Each group had 6 replicates of 12 chicks. The size of the test chicken cage (length×width×height) was 60 cm×40 cm×40 cm, and 4 chicks were raised in each cage. A LED light lamp was hung on the top of the cage. White (439.20 and 568.10 nm), blue (454.40 nm), and green (517.30 nm) LED lamps (purchased from NVC Co., Ltd., Huizhou, China) were used as light sources. The illumination intensity of the four corners, the center and the trough of the cage was measured with TES-1339 illuminometer (TES Electrical Electronic Corp, Taipei, China), and the average value was calculated as the illumination intensity of the experimental design. The light intensity for days 8 to 42 of the experiment was set at 3 levels: 5, 10, and 15 lx.

During the experiment, all chickens were raised in three-layer cages in the temperature-controlled room with a 23 h light: 1 h dark lighting program, ate and drank *ad libitum*. Ambient temperature was 32°C at 8 days and then gradually reduced by 2°C per week to a final temperature of 22°C. The experimental period was 35 days. The broilers in all groups were fed with compound feed purchased from Shandong New Hope Liuhe Group Co., Ltd., Qingdao, China.

### Sample collection

On the 42nd day of the experiment, after 12 h of starvation, 12 birds (2 bird per replicate) were randomly selected from each treatment group. Subsequently, samples from the right breast muscle, parts of the left pectoralis major muscle and right thigh muscle were immediately excised and stored at 4°C for determination of meat quality and nutrition composition. Blood samples were centrifuged at 3,500 r/min for 10 min. Sera were separated and stored at −20°C for the detection of serum antioxidant capacity.

### Slaughter performance

The weight of broilers after plucking and bloodletting was taken as slaughter weight (SW) and after removal of head, foot, and viscera was taken as eviscerated weight (EW). Dressing percentage (DP), semi-eviscerated weight percentage (SEWP), and EWP were calculated by SW, SEW, and EW/BW. Eviscerated yield was calculated as the percentages of BW. Breast and thigh muscle were separated and weighed. Breast muscle percentage (BMP) and thigh muscle percentage (TMP) were calculated as the percentages of EW, according to the method described by Ding et al [15].

### Meat quality determination

The breast after slaughter were taken to detect the muscle pH, flesh color, shear force (SF), water-loss percentage (WLP). At 45 min and 24 h postmortem, the pH values of collected breast muscle were measured by direct insertion of an electrode (PHSJ-4F; INESA Scientific Instrument Co., Ltd., Shanghai, China). At 45 min after slaughter, the lightness (L*), redness (a*), and yellowness (b*) of breast muscle were measured by CR-400 (Konica Minolta Holdings, Inc., Tokyo, Japan) automatic color difference meter, according to the method described by Hu et al [16]. At 45 min after slaughter, a long strip of breast muscle sample (without tendon, fat, and muscle membrane) with a length of 3 cm, a width of 1 cm and a thickness of 1cm was trimmed. The SF was measured by C-LM3 (College of engineering, Northeast Agricultural University, Harbin, China) digital display muscle tenderness instrument, according to the method described by Gao et al [17]. Accurately weighted the fresh sample of breast muscle at the same part, placed it in three layers of filter paper with good water absorption at the top and bottom, pressurized 500 N with WW-3 strain type unconfined pressure gauge (Nanjing Soil Instrument Factory Co., Ltd., Nanjing, China) and kept it for 3min, removed the pressure, weighted the pressed meat sample, according to the method described by Hu et al [18].

### Inosinic acid and related nucleotides

Take 5 g breast muscle sample, remove the visible fat, put it into a 50 mL plastic homogenization tube, add 15 mL 5% perchloric acid solution, beat it into a slurry with a FJ200-SH high-speed tissue homogenizer (Shanghai Specimen and Model Factory, Shanghai, China) in ice bath, wash the homogenizer with 10 mL 5% perchloric acid and 5 mL deionized water, combine the homogenization solution, centrifuge at 4,000 r/min for 10 min, and filter the supernatant into a 100 mL beaker through medium speed quantitative filter paper, The precipitation is then shaken with 15 mL 5% perchloric acid solution for 5 min, centrifuged at 3,500 r/min for 10 min, filtered and combined with the filtrate. Adjust the pH to 6.5 with 1% sodium hydroxide solution, transfer it to a 100 mL volumetric flask, fix the volume with deionized water. The samples were filtered into the automatic vial and then used Allent 1100 (Agilent Technologies Inc., Santa Clara, CA, USA) for high performance liquid chromatography (HPLC).

### Main nutrients in muscle

Placed about 50 g of thigh muscle sample in a glass dish, weighed it, put it in FDU-1200 freeze dryer (Tokyo Rikakikai Co., Ltd., Tokyo, Japan) with −45°C for 48 h, and then reweighed to calculate the moisture percentage. Then, the dried samples were made into powder with FW80 (Tianjin Taisite Instrument Co., Ltd., Tianjin, China) for the analysis the contents of dry matter (DM), crude protein (CP), crude fat (CF) and intramuscular fat (IMF). The contents of DM, CP, and CF were respectively determined with GB5009.3-2016 (China), GB5009.5-2016 (China), and GB5009.6-2016 (China). The content of IMF was determined by Soxhlet extraction method.

### Serum antioxidant capacity

The activities of glutathione peroxidase (GSH-Px), superoxide dismutase (SOD), and malondialdehyde (MDA) in serum were detected by using a tested kit according to the corresponding protocols provided by Nanjing Jiancheng Bioengineering Institute (Nanjing, China).

### Statistical analysis

Replicate was considered as the experimental unit. Data were analyzed as a 3×3 (light colors×light intensities) factorial arrangement of treatments by two-way analysis of variance with a model including the main effects of light colors, light intensities and their interaction using the general linear model procedure of the SPSS (version 22.0 for Windows; SPSS Inc., Chicago, IL, USA). Differences among treatments were examined using Tukey’s multiple range tests and were considered to be significant when p<0.05 or p<0.01. Data were presented as means with their pooled standard errors.

## RESULTS

### Slaughter performance

Table 1 indicated that the SEWP of 5 lx white group was extremely significantly higher than that of 15 lx white group (p<0.01). The EWP of 15 lx green group was significantly higher than that of 10 lx white group (p<0.05). There was no significant difference in DP, BMP, and TMP among the groups (p>0.05). Compared with blue and green illumination, the indexes of DP, SEWP, BMP, and TMP of broilers in white illumination were improved, and compared with other two light intensities, 10 lx intensity increased BMP and TMP of broilers. The effect of light color on TMP was extremely significant (p<0.01). Light intensity has a significant effect on SEWP and EWP (p<0.05). The interaction effect of light color and light intensity has no significant effect on DP (p = 0.053), SEWP (p = 0.057), EWP (p = 0.451), BMP (p = 0.220), and TMP (p = 0.066).

### Meat quality determination

As shown in Table 2, the WLP in 5 lx green group was extremely significantly higher than that in 10 lx white group (p<0.01). There was no significant difference in L value, a value, b value, SF, and pH value among the groups (p>0.05). Compared with the other two light color groups, the white light group increased L* value and SF, the green light group increased a*, b* value, and WLP, and the blue light group increased pH value. Compared with the other two light intensity groups, 10 lx illumination increased the L*, a*, and b* values of meat, and 15 lx illumination increased SF and pH values. Light color had a very significant effect on WLP (p<0.01), and light intensity had a significant effect on L value (p<0.05) and WLP (p<0.01). The interaction effect of light color and light intensity had no significant effect on L* value (p = 0.726), a* value (p = 0.609), b* value (p = 0.292), WLP (p = 0.461), SF (p = 0.952), pH_45 min_ (p = 0.970), and pH_24 h_ (p = 0.929).

### Inosinic acid and related nucleotides

It can be seen from Table 3 that the content of hypoxanthine (Hx) in blue groups were very significantly lower than that in white and green groups (p<0.01). There was no significant difference in the contents of inosine monophosphate acid (IMP), adenosine diphosphate (ADP), adenosine monophosphate (AMP), and inosine (HxR) among the groups (p>0.05). Compared with the other two light color groups, the white light group increased the content of IMP, ADP, AMP, Hx, and HxR. Compared with the other two light intensity groups, 10 lx illumination increased the IMP content of meat, and 15 lx illumination increased the HxR content of meat. Light color had a significant effect on content of ADP (p<0.05) and Hx (p<0.01). The interaction of light color and light intensity had no significant effect on the contents of IMP (p = 0.732), ADP (p = 0.965), AMP (p = 0.249), Hx (p = 0.985) and HxR (p = 0.101).

### Main nutrients in muscle

In Table 4, there was no significant difference in contents of DM, CP, CF, and IMF among the groups (p>0.05). Compared with the other two illumination color groups, green illumination increased DM and CP contents and decreased IMF content in muscle. Light color had a significant effect on the content of CP (p<0.01), and the interaction effect of light color and light intensity had no significant effect on the contents of DM (p = 0.556), CP (p = 0.998), CF (p = 0.936), and IMF (p = 1.000).

### Serum antioxidant capacity

Table 5 showed that the content of MDA in 15 lx blue group was significantly higher than that in 10 lx green group (p< 0.05). There was no significant difference in the content of SOD and GSH-Px among the groups (p>0.05). Compared with the other two illumination intensity groups, 10 lx illumination increased the serum content of SOD and GSH-Px and decreased the serum MDA content. Light intensity had a significant effect on the contents of SOD (p<0.05), MDA (p<0.01), and GSH-Px (p<0.01). The interaction of light color and light intensity had no significant effect on the content of SOD (p = 0.802), MDA (p = 0.784), and GSH-Px (p = 0.668).

## DISCUSSION

Carcass performance index is one of the important indexes to measure poultry production efficiency. At present, there are many reports on the effects of different light source colors on the slaughter performance of broilers, but the results are not consistent. It has been reported that there is no significant difference between color and intensity of light on carcass indexes such as breast muscle weight, thigh muscle weight and abdominal fat weight of broilers [19,20]. The cold-white (6,065K) improved the final live BW and the yield from the breast muscle tenders (Pectoralis minor) without compromising the measured welfare indicators [5]. It is reported that the BW of broilers raised under blue light may have leg problems [21], but the BW of broilers in our test at slaughter day may have been too small to experience leg problems. Therefore, the effectiveness of the continuous use of blue light source needs further experimental research. Ross 708 broilers were raised in the environmental control cabin and illuminated with LED warm light, LED cold light and incandescent lamp, the results showed that there was no significant difference between LED warm light and LED cold light on broiler carcass weight, DP, BMP, and TMP [9]. Our results showed that the BMP and TMP of 10 lx white light group were significantly higher than those of other groups, and the EWP of 15lx light intensity was significantly higher than that of other light intensity groups. Olanrewaju et al [10] indicated that the difference of live weight and carcass weight were only between chickens raised under cool-poultry specific filtered LED (Cool-PSF-LED) and with those raised under incandescent (ICD) the light intensity had no effect. Halevy et al [22] pointed out that green light can promote muscle growth, especially chest muscle, which is basically consistent with the results of this test. In addition, it is also reported that green light promotes the growth of broiler muscle in the early stage of growth, and blue light promotes the growth of broiler muscle in the later stage of growth [23]. Our results showed that the CF of white light group was lower than that of other groups, indicating that the fat deposition ability of broilers in white light environment was low. This may be because chickens are quiet in monochromatic light, but more active in white light and have a large amount of exercise [24].

Meat quality is not only an important economic characteristic but also a comprehensive evaluation feature of animal husbandry production [25]. Too long light is not conducive to the brightness of chicken color, light affects meat color by adjusting the deoxygenation of iron brin group of oxygenated myoglobin [26]. The pHu can be used as the judgment index of water holding capacity and flesh color [21]. In this study, the pHu of broilers in 15 lx light group was the highest. It may be because the activity of broilers in the 15 lx light group is the largest, most of the glycogen stored in thigh muscles is used for exercise consumption, and the storage is relatively small. Ross 708 broiler chickens were raised on the ground with energy-saving lamps of 5 lx and 20 lx light intensity, neutral light-emitting diodes, special filter LEDs for cold light poultry and incandescent lamps, the results showed that the light intensity had no significant effect on the weight of pectoralis major and pectoralis minor muscles [27]. Some research results show that the fat deposition ability of yellow feather broilers in white light environments is low, which may be due to chickens being more active and exercise more in white light [24]. This result showed that the CF content of breast meat in white light group was lower than that in other light groups. IMP is a flavor-enhancing substance, which plays a critical role in the umami taste of the muscle, making the content of IMP an important umami taste indicator [28]. At present, the research on the effect of muscle IMP content focuses on genetic factors, feeding methods, nutritional factors and the age at slaughter, and there is no research report on the influence of light environment test on it, although the muscle adenosine triphosphate (ATP) content was measured in this test, the content was not detected, which may be due to the rapid decomposition of ATP.

The SOD and GSH-Px are the main antioxidant enzymes in cells, their activity reflects the ability of scavenging free radicals [29]. The MDA is one of the final products of polyunsaturated fatty acids peroxidation in the cells. Its level is commonly known as a marker of oxidative stress and the antioxidant status [30,31]. Zheng et al [32] reported that low light intensity of 5 lx significantly enhanced activity of GSH-Px of 21 d broiler chickens in serum. Broilers reared under intermittent lighting (17 L:3 D:1 L:3 D) and 10 lx of light intensity can improve antioxidant status and immune function [33]. The results showed that the contents of SOD and GSH-Px in 5 lx and 10 lx irradiation groups were higher than those in 15 lx irradiation group, and the content of MDA in 5 lx and 10 lx irradiation groups was lower than that in 15 lx irradiation group, which was consistent with the reported results.

## CONCLUSION

Using white LED light with 10 lx light intensity can significantly improve the chicken meat quality of caged Cobb broilers, improve the content of inosine acid in chicken breast and enhance the antioxidant capacity of the body. We recommend that white feather broiler farms use white LED lights with 10 lx intensity for 8 to 42 days.

## Figures and Tables

**Table 1:** Effects of LED light sources on slaughter performance of broilers (%)^1)^

Items	Light intensity (lx)	White light	Green light	Blue light	SEM	p-value		

						Color	Intensity	Color×intensity^2)^
DP	5	93.47	93.11	92.17	0.17	NS	NS	NS
	10	93.05	92.07	92.31				
	15	91.91	92.15	93.35				
SEWP	5	83.93^A^	83.05^AB^	82.24^AB^	0.16	NS	^*^	NS
	10	82.55^AB^	82.38^AB^	82.78^AB^				
	15	81.51^B^	81.82^AB^	82.51^AB^				
EWP	5	70.87^ab^	70.90^ab^	69.96^ab^	0.16	NS	^*^	NS
	10	69.19^b^	70.35^ab^	69.90^ab^				
	15	71.04^ab^	71.29^a^	70.46^ab^				
BMP	5	30.48	27.96	29.40	0.34	NS	NS	NS
	10	30.75	30.26	29.38				
	15	29.87	31.02	27.92				
TMP	5	25.59	23.37	22.73	0.27	^**^	NS	NS
	10	25.94	24.03	23.51				
	15	24.59	22.95	25.40				

LED, light-emitting diode; SEM, standard error of the mean; DP, dressing percentage; SWP, semi-eviscerated weight percentage; EWP, eviscerated weight percentage; BMP, breast muscle percentage; TMP, thigh muscle percentage.

1) Data represent the means from 6 replicates per treatment.

2) Color×intensity, interaction between color and intensity.

a,b Means with different superscripts within the same item are significantly different (p<0.05).

A,B Means with different superscripts within the same item are extremely significantly different (p<0.01).

* Means p<0.05,

** means p<0.01,

NS, means no significant difference.

**Table 2:** Effects of LED light sources on meat quality of broilers^1)^

Items	Light intensity (lx)	White light	Green light	Blue light	SEM	p-value		

						Color	Intensity	Color×intensity^2)^
L*	5	63.40	60.67	63.42	0.70	NS	^*^	NS
	10	66.05	66.49	66.32				
	15	65.07	60.27	61.85				
a*	5	4.55	4.57	4.70	0.04	NS	NS	NS
	10	4.70	4.86	4.62				
	15	4.58	4.72	4.61				
b*	5	5.48	5.75	5.96	0.08	NS	NS	NS
	10	6.21	5.97	5.80				
	15	5.64	5.98	5.53				
WLP (%)	5	33.62^AB^	37.72^A^	36.65^AB^	0.35	^**^	^**^	NS
	10	32.74^B^	34.40^AB^	33.88^AB^				
	15	33.64^AB^	35.17^AB^	34.21^AB^				
SF (kg/f)	5	3.86	3.86	3.88	0.01	NS	NS	NS
	10	3.85	3.84	3.84				
	15	3.88	3.86	3.86				
pH_45 min_	5	5.48	5.48	5.47	0.01	NS	NS	NS
	10	5.45	5.46	5.48				
	15	5.48	5.49	5.52				
pH_24 h_	5	5.06	5.07	5.05	0.01	NS	NS	NS
	10	5.04	5.05	5.07				
	15	5.06	5.08	5.10				

LED, light-emitting diode; SEM, standard error of the mean; WLP, water-loss percentage; SF, shear force; pH_45 min_, muscle pH value at 45 min post mortem; pH_24 h_, muscle pH value at 24 h post mortem.

1) Data represent the means from 6 replicates per treatment.

2) Color×intensity, interaction between color and intensity.

A,B Means with different superscripts within the same item are extremely significantly different (p<0.01).

* Means p<0.05,

** means p<0.01,

NS means no significant difference.

**Table 3:** Effects of LED light sources on IMP and its related nucleotide acids in chicken (mg/g)^1)^

Items	Light intensity (lx)	White light	Green light	Blue light	SEM	p-value		

						Color	Intensity	Color×intensity^2)^
IMP	5	1.05	0.97	0.92	0.03	NS	NS	NS
	10	1.06	0.91	1.07				
	15	0.99	0.72	0.83				
ADP	5	0.21	0.16	0.20	0.01	^*^	NS	NS
	10	0.18	0.15	0.19				
	15	0.19	0.16	0.19				
AMP	5	0.07	0.05	0.06	0.00	NS	NS	NS
	10	0.06	0.06	0.06				
	15	0.06	0.06	0.04				
Hx	5	0.19^A^	0.19^A^	0.10^B^	0.01	^**^	NS	NS
	10	0.20^A^	0.19^A^	0.11^B^				
	15	0.20^A^	0.19^A^	0.11^B^				
HxR	5	1.65	1.93	2.03	0.08	NS	NS	NS
	10	2.12	1.80	1.30				
	15	1.77	2.10	1.44				

LED, light-emitting diode; IMP, inosine monophosphate acid; SEM, standard error of the mean; ADP, adenosine diphosphate; AMP, adenosine monophosphate; Hx, hypoxanthine; HxR, inosine.

1) Data represent the means from 6 replicates per treatment.

2) Color×intensity, interaction between color and intensity.

A,B Means with different superscripts within the same item are extremely significantly different (p<0.01).

* Means p<0.05,

** means p<0.01,

NS means no significant difference.

**Table 4:** Effect of LED light sources on chicken nutrient contents of broilers (%)^1)^

Items	Light intensity (lx)	White light	Green light	Blue light	SEM	p-value		

						Color	Intensity	Color×intensity^2)^
DM	5	27.46	28.00	27.19	0.16	NS	NS	NS
	10	26.47	27.47	27.61				
	15	27.48	27.60	27.35				
CP	5	68.97	69.62	68.87	0.12	^**^	NS	NS
	10	68.98	69.80	68.89				
	15	68.79	69.67	68.80				
CF	5	31.76	32.58	33.59	0.21	NS	NS	NS
	10	31.78	32.95	32.75				
	15	32.47	31.75	32.59				
IMF	5	3.74	3.72	3.74	0.03	NS	NS	NS
	10	3.75	3.75	3.72				
	15	3.74	3.74	3.74				

LED, light-emitting diode; SEM, standard error of the mean; DM, dry matter; CP, crude protein; CF, crude fat; IMF, intramuscular fat.

1) Data represent the means from 6 replicates per treatment.

2) Intensity×color, interaction between intensity and color.

* Means p<0.05,

** means p<0.01,

NS means no significant difference.

**Table 5:** Effect of LED light sources on serum antioxidant index of broilers^1)^

Items	Light intensity (lx)	White light	Green light	Blue light	SEM	p-value		

						Color	Intensity	Color×intensity^2)^
SOD (U/mL)	5	200.43	204.47	203.24	1.08	NS	^*^	NS
	10	208.99	207.91	208.20				
	15	199.51	201.68	205.55				
MDA (nmol/mL)	5	3.36^ab^	3.21^ab^	3.42^ab^	0.04	NS	^**^	NS
	10	3.33^ab^	3.13^b^	3.23^ab^				
	15	3.48^ab^	3.45^ab^	3.70^a^				
GSH-Px (U/mL)	5	547.49	541.26	548.25	2.21	NS	^**^	NS
	10	564.88	569.28	558.74				
	15	555.14	547.45	546.85				

LED, light-emitting diode; SEM, standard error of the mean; SOD, superoxide dismutase; MDA, malondialdehyde; GSH-Px, glutathione peroxidase.

1) Data represent the means from 6 replicates per treatment.

2) Color×intensity, interaction between color and intensity.

a,b Means with different superscripts within the same item are significantly different (p<0.05).

* Means p<0.05,

** means p<0.01,

NS means no significant difference.
